# Cause of Death in Patients with Oropharyngeal Carcinoma by Human Papillomavirus Status: Comparative Data Analysis

**DOI:** 10.2196/47579

**Published:** 2023-08-29

**Authors:** Dong-Dong Zhang, Min Lei, Yue Wang, Pei-Ji Zeng, Yong-Jun Hong, Cheng-Fu Cai

**Affiliations:** 1 Department of Otolaryngology-Head and Neck Surgery School of Medicine, Zhongshan Hospital Xiamen University Xiamen China; 2 School of Medicine Xiamen University Xiamen China; 3 Xiamen Key Laboratory of Otolaryngology Head and Neck Surgery Xiamen Xiamen China; 4 Department of Otorhinolaryngology Head and Neck Surgery Xiamen University Xiamen China; 5 Department of Otolaryngology Head and Neck Surgery Fujian Medical University Xiamen China; 6 College of Otorhinolaryngology Head and Neck Surgery Xiamen Haicang Hospital Xiamen China

**Keywords:** oropharyngeal carcinomas, human papillomavirus, causes of death, survival, competing mortality, human papillomavirus, comparative data, analysis, cell death, carcinoma, oropharyngeal carcinoma, surveillance, database, human papillomavirus-related tumor

## Abstract

**Background:**

The incidence of oropharyngeal squamous cell carcinomas (OPSCC) has increased in recent decades, and human papillomavirus (HPV) infection is the main cause of OPSCC. The data regarding causes of death (CODs) are vitally important in informing follow-up strategies and revising treatment strategies to deal with any possible preventable treatment-related COD. However, limited studies have assessed the competing COD by HPV status in patients with OPSCC.

**Objective:**

We aimed to analyze the distribution of the competing COD according to HPV status in OPSCC.

**Methods:**

We retrospectively included stage I-IVB patients with OPSCC from the Surveillance, Epidemiology, and End Results database between 2010 and 2015. The association between HPV status and head and neck cancer–specific mortality (HNCSM), second primary cancer mortality (SPCM), and noncancer-caused mortality (NCCM) were analyzed. The chi-square test, Kaplan-Meier analysis, and Fine and Gray model were used for statistical analysis.

**Results:**

We included 5852 patients in this study and 73.2% (n=4283) of them had HPV-related tumors. A total of 1537 (26.3%) patients died, including 789 (51.3%), 333 (21.7%), and 415 (27%) patients who died from head and neck cancer, second cancer, and noncancer causes, respectively. The 5-year HNCSM, SPCM, NCCM, and overall mortality were 14.7%, 6.5%, 7.7%, and 26.4%, respectively. Those with HPV-positive disease had a lower cumulative incidence of HNCSM (subdistribution hazard ratio [sHR] 0.362, 95% CI 0.315-0.417; *P*<.001), SPCM (sHR 0.400, 95% CI 0.321-0.496; *P*<.001), and NCCM (sHR 0.460, 95% CI 0.378-0.560; *P*<.001) than those with HPV-negative disease. The 5-year risk of HNCSM was 26.9% and 10.7% in those with HPV-negative and HPV-positive disease, respectively (*P*<.001). The 5-year risk of SPCM was 12.4% and 4.6% in those with HPV-negative and HPV-positive disease, respectively (*P*<.001). The 5-year risk of NCCM of death was 13.7% and 5.8% in those with HPV-negative and HPV-positive disease, respectively (*P*<.001). Using the Fine and Gray competing-risks model, our results show that those with HPV-negative tumors had a significantly higher risk of HNCSM (*P*<.001), SPCM (*P*<.001), and NCCM (*P*<.001) than those with HPV-negative tumors.

**Conclusions:**

HPV-positive OPSCC has a lower NCSM, SPCM, and NCCM as compared to those with HPV-negative OPSCC. HPV positivity is a favorable prognostic factor in the context of overcoming cancer as well as in terms of reducing the risk of other CODs in OPSCC. Our finding supports the need to tailor patient follow-up based on the HPV status of patients with OPSCC.

## Introduction

Human papillomavirus (HPV) is the most prevalent sexually transmitted infection globally and is associated with both anogenital and oropharyngeal cancers. It is estimated that over 80% of individuals will be affected by HPV at some point in their lifetime [1]. Like other types of head and neck squamous cell carcinomas (HNSCCs), oropharyngeal squamous cell carcinoma (OPSCC) has been historically associated with alcohol and tobacco consumption. However, infection with carcinogenic HPV infection has emerged as an important risk factor that has driven an increase in the incidence of OPSCC. In recent decades, OPSCC has been one of the malignant tumors with rapidly rising incidences in Western countries [2-5]. In Italy, the incidence of HPV-related OPSCC increased from 16.7% in 2000-2006 to 46.1% in 2013-2018 [6]. Moreover, HPV now accounts for 71% and 51.8% of all OPSCCs in the United States and the United Kingdom, respectively [7-9]. The etiological relation with OPSCC by HPV status was also found in the Asian population [10,11]. The existing evidence shows that HPV status impacts the prognosis and treatment decision-making of OPSCC [12,13]. Patients with HPV-positive disease had a significantly better prognosis than those with HPV-negative disease [12,13]. Moreover, several studies also explored the treatment de-escalation for HPV-positive OPSCC [14,15]. These differences have a direct effect on clinical trials that will determine treatment therapeutic standards in the future clinical practice of OPSCC.

As survivorship from OPSCC continues to increase in recent decades [16-18], the main participants in the medical field should identify the patients with OPSCC with the highest risk of dying as well as the risk of their specific cause of death (COD). In addition to HPV, smoking and alcohol use are also closely related to the development of OPSCC, which may cause competing mortality in this population. In 2 studies from the Netherlands and the US cancer registry showed that the survival outcomes of head and neck cancer (HNC) were inferior to that of a healthy population of similar gender and age [19,20]. Smoking and alcohol use are the causative factors for cardiopulmonary diseases and second primary tumors. A previous Surveillance, Epidemiology, and End Result (SEER) study showed that life expectancy for patients with HNC was significantly shorter than expected after excluding the COD related to HNC [21]. The other COD may contribute to the differences in the survival of patients with HNC [22,23]. This information is vitally important in informing follow-up strategies and revising treatment strategies to deal with any possible preventable treatment-related COD.

Because HPV-positive patients with OPSCC are usually younger and consume less alcohol and tobacco, the risk of cardiopulmonary disease and secondary primary tumor in this population may be lower [24,25]. The differences in survival rates for patients with HNSCC can primarily be attributed to an unequal burden of comorbidities associated with tobacco and alcohol use, treatment-related sequelae, and a high risk for the development of second primary cancer. Moreover, the surveillance for HPV-related tumors is similar to HPV-negative tumors in the current guidelines. Given the rapid rise in the incidence of potentially HPV-related OPSCC, it is crucial to provide a more comprehensive description of the COD in OPSCC. Understanding the COD by different HPV status in OPSCC could bring even more positive impact on public health, potentially impacting the follow-up strategies and revising treatment strategies while reducing treatment-related morbidity. However, limited studies have assessed the competing COD by HPV status in patients with OPSCC [26]. The objective of this study was to analyze the differences in the competing COD depending on HPV status in OPSCC.

## Methods

### Patient Selection

**Figure 1:**
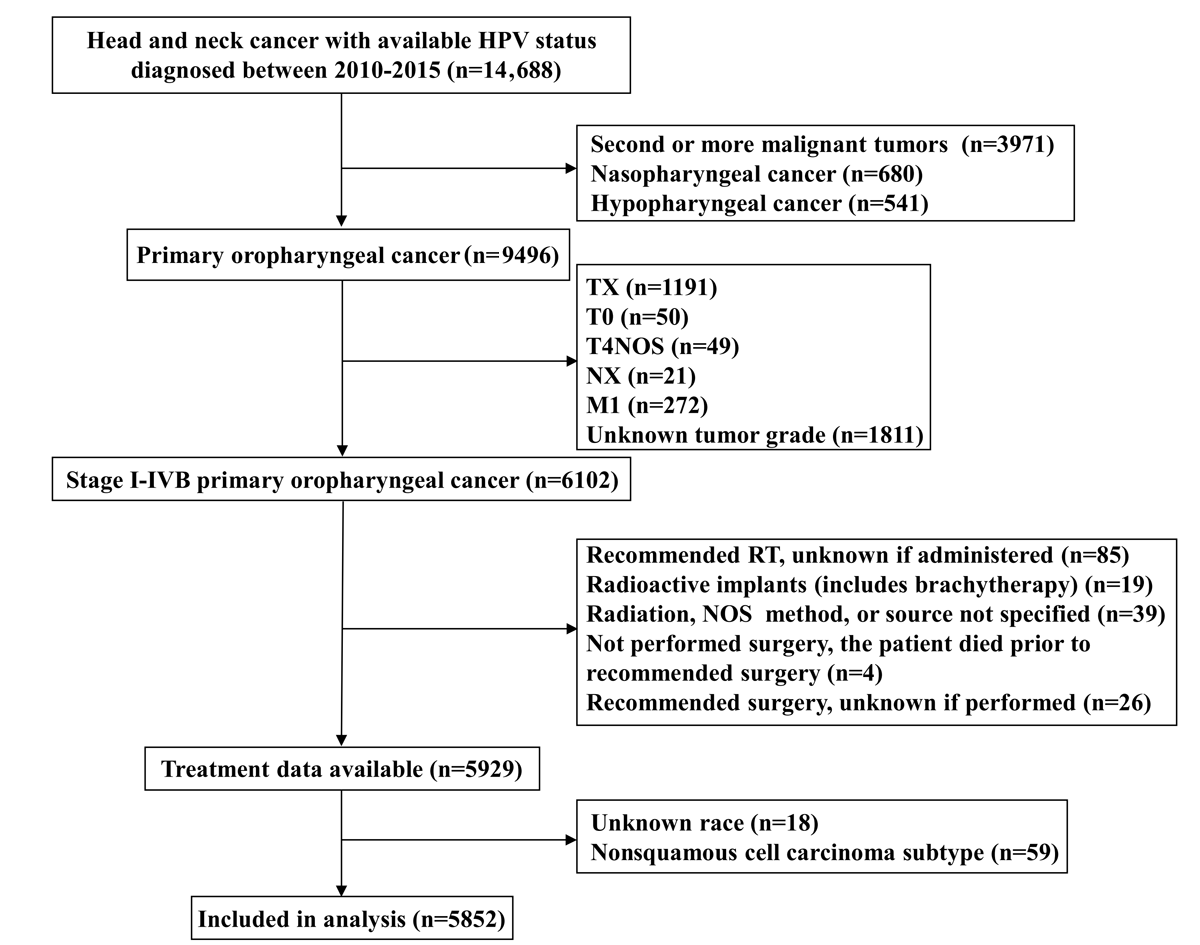
Flow diagram of this study’s cohort. HPV: human papillomavirus; M: metastatic stage; N: nodal stage; NOS: not otherwise specified; NX: unknown nodal stage; RT: radiotherapy; T: tumor stage; TX: unknown tumor stage.

The National Cancer Institute SEER database collects population-level data on approximately 28% of the US population from 18 registries with the morbidity, mortality, and disease status of millions of patients with cancer [27]. We used the SEER databases to study patients diagnosed with OPSCC between 2010 and 2015 (International Classification of Disease for Oncology ICD-O C019, C090, C091, C098-C104, C108, C109, C142; histology codes 8073-8079 and 8083-8084). Registrars for the SEER database were required to enter HPV status beginning in 2010 and we have obtained HPV-related variables through the use of a data user agreement. The data of the retrospective cohort in the present study were obtained from the SEER HPV database, which includes the demographic data, clinicopathological characteristics, treatment, HPV status, and follow-up of patients with HNSCC [27]. We included patients who met the following criterion: (1) diagnosed between 2010 and 2015; (2) diagnosed with stage I-IVB OPSCC according to the 7th American Joint Committee on Cancer (AJCC) staging system; (3) available data regarding HPV status, race, tumor grade, AJCC staging, tumor stage, nodal stage, and metastatic stage; and (4) available data on surgery, radiotherapy, and chemotherapy. Patients with unknown HPV status, second or more malignant tumors, distant stage, and treated with radioactive implants or radioisotopes were excluded. The patient selection flowchart is listed in Figure 1.

### Ethics Approval

The SEER database is a public database, and we have obtained permission to access the database (approval number 18275-Nov2021). The present study did not require ethical approval by the institutional review board of the Zhongshan Hospital, Xiamen University, because of the nonidentifying information in the SEER database. The requirement for informed consent was waived because all data used in this study were deidentified.

### Data Collection

The following variables were included: age, gender, race, tumor grade, HPV status, AJCC 7th staging, and the receipt of treatment. HPV status was determined by p16 immunohistochemistry, tissue polymerase chain reaction, in situ hybridization, real-time polymerase chain reaction for E6/7 RNA, or in situ hybridization for E6/7 RNA [28]. The primary outcomes were HNC-specific mortality (HNCSM), second primary cancer mortality (SPCM), and noncancer-caused mortality (NCCM). Any COD due to HNC was encoded as HNCSM. Events for SPCM were death from the second primary cancer. Events for NCCM were death from noncancer diseases. Our secondary outcome was overall mortality (OM). OM was defined as death from all causes.

### Statistical Analysis

Categorical variables were represented as absolute values with percentages, and the chi-square test was performed for the comparison between patients who were HPV-negative and HPV-positive. Kaplan-Meier analysis was used to generate the survival curves to compare differences in survival between groups over time, and the equality of these curves was compared by the log-rank test. Due to the competitive relationship among multiple outcomes in clinical survival data, the traditional Kaplan-Meier analysis will misjudge the cumulative mortality rate [29]. Therefore, competing-risks analyses using the Fine and Gray model were used to estimate the effects of covariates on HNCSM, SPCM, and NCCM. We conducted stratum-specific analyses to estimate the effects of HPV status on HNCSM, SPCM, and NCCM in accordance with gender, age, race, AJCC staging, grade, surgery, radiotherapy, and chemotherapy. All statistical analyses were conducted using SPSS (version 22.0; IBM Corp) and Stata/SE (version 14; StataCorp). A *P* value <.05 was considered statistically significant.

## Results

### Patient Baseline Characteristics

From 2010 to 2015, there were 14,688 cases of HNC recorded with the HPV status in the SEER HPV database; 9496 of these cases were oropharyngeal cancer and 5852 of these cases were stage I-IVB OPSCC and had treatment data available. In this cohort (n=5852; Table 1), 4283 patients (73.2%) had HPV-positive OPSCC and 1569 patients (26.8%) had HPV-negative OPSCC. Of these patients, 84.1% (n=4922), 89.2% (n=5220), and 69.9% (n=4088) were male, White, and had stage T4 disease, respectively. Regarding treatment, 89.1% (n=5216), 71.3% (n=4174), and 55% (n=2626) of patients received radiotherapy, chemotherapy, and surgery, respectively.

**Table 1:** Patient baseline characteristics.

Variables		Patients, n	HPV^a^-negative, n (%)	HPV-positive, n (%)	*P* value
**Gender**					<.001
	Male	4922	1213 (77.3)	3709 (86.6)	
	Female	930	356 (22.7)	574 (13.4)	
**Age (years)**					<.001
	<50	840	207 (13.2)	633 (14.8)	
	50-64	3425	847 (54)	2578 (60.2)	
	≥65	1587	515 (32.8)	1072 (25)	
**Race**					<.001
	White	5220	1288 (82.1)	3932 (91.8)	
	Black	414	212 (13.5)	202 (4.7)	
	Other	218	69 (4.4)	149 (3.5)	
**Grade**					<.001
	Well differentiated	233	113 (7.2)	120 (2.8)	
	Moderately differentiated	2240	754 (48.1)	486 (34.7)	
	Poorly or undifferentiated	3379	702 (44.7)	2677 (62.5)	
**AJCC^b^ stage**					<.001
	I	218	93 (5.9)	125 (2.9)	
	II	384	108 (6.9)	276 (6.4)	
	III	1162	342 (21.8)	820 (19.1)	
	IVA and IVB	4088	1026 (65.4)	3062 (71.5)	
**T^c^ stage**					<.001
	T1	1630	360 (22.9)	1270 (29.7)	
	T2	2322	527 (33.6)	1795 (41.9)	
	T3	1057	342 (21.8)	715 (16.7)	
	T4	843	340 (21.7)	503 (11.7)	
**N^d^ stage**					<.001
	N0	935	338 (21.5)	597 (13.9)	
	N1	1096	326 (20.8)	770 (18)	
	N2	3562	836 (53.3)	2726 (63.6)	
	N3	259	69 (4.4)	190 (4.4)	
**Surgery**					<.001
	No	3226	964 (61.4)	2262 (52.8)	
	Yes	2626	605 (38.6)	2021 (47.2)	
**Radiotherapy**					<.001
	No	636	228 (14.5)	408 (9.5)	
	Yes	5216	1341 (85.5)	3875 (90.5)	
**Chemotherapy**					.02
	No	1678	487 (31)	1191 (27.8)	
	Yes	4174	1082 (69)	3092 (72.2)	
**Causes of death (N=1537)**					.50
	Head and neck cancer	789	378（52.6）	411 (50.2)	
	Second cancer	333	156 (21.7)	177 (21.6)	
	Noncancer cause disease	415	184 (25.6)	231 (28.2)	

^a^HPV: human papillomavirus.

^b^AJCC: American Joint Committee on Cancer.

^c^T: tumor stage.

^d^N: nodal stage.

**Figure 2:**
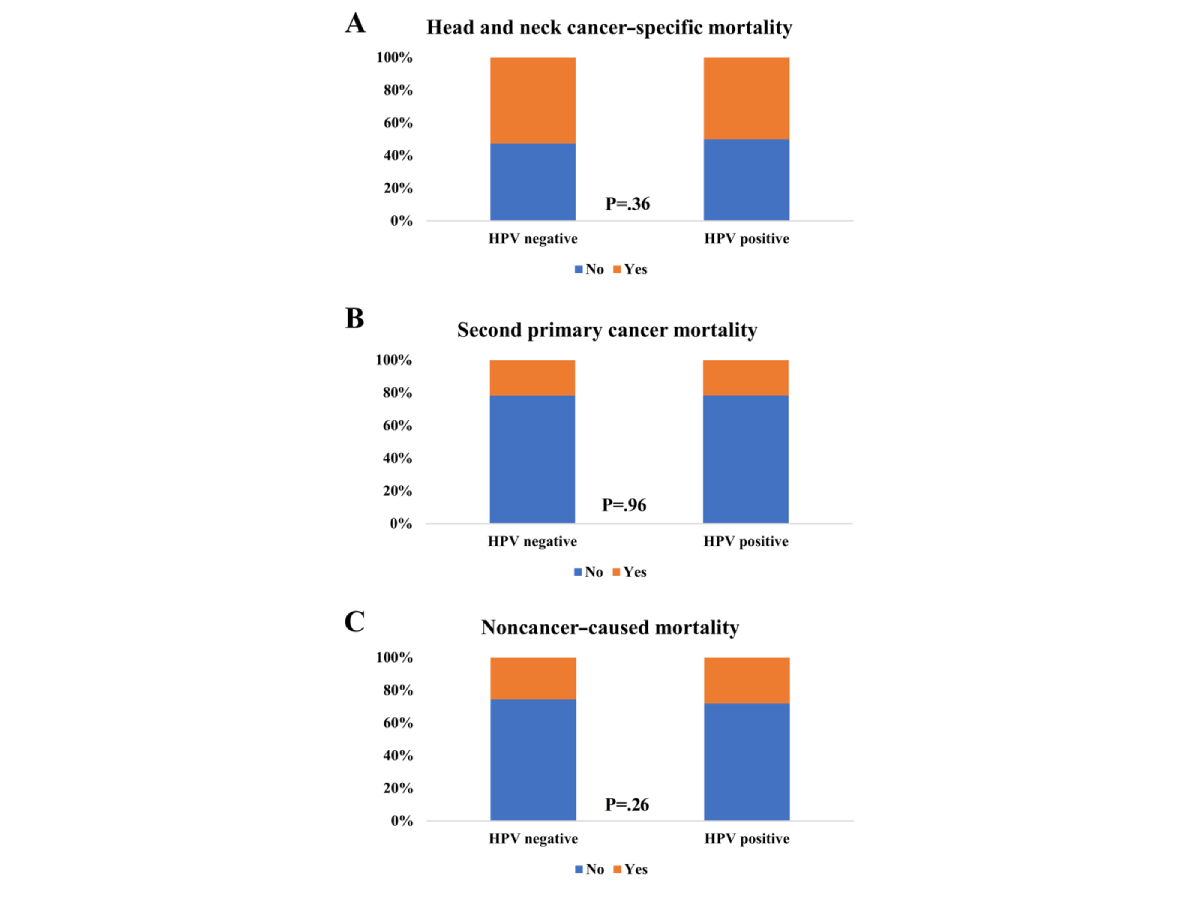
The distributions in (A) head and neck cancer–specific mortality, (B) second primary cancer mortality, and (C) noncancer-caused mortality according to human papillomavirus status. HPV: human papillomavirus.

Patients with HPV-positive disease were more likely to be male, aged 50-64 years, White, have higher tumor grade, have early tumor stage, have advanced nodal stage, and have stage IV disease (all *P*<.001). Moreover, those with HPV-positive disease were more likely to receive surgery (*P*<.001) and radiotherapy (*P*=.015; Table 1). Similar distributions in HNCSM (*P*=.36), SPCM (*P*=.96), and NCCM (*P*=.26) were found according to HPV status (Figure 2).

### Survival

**Figure 3:**
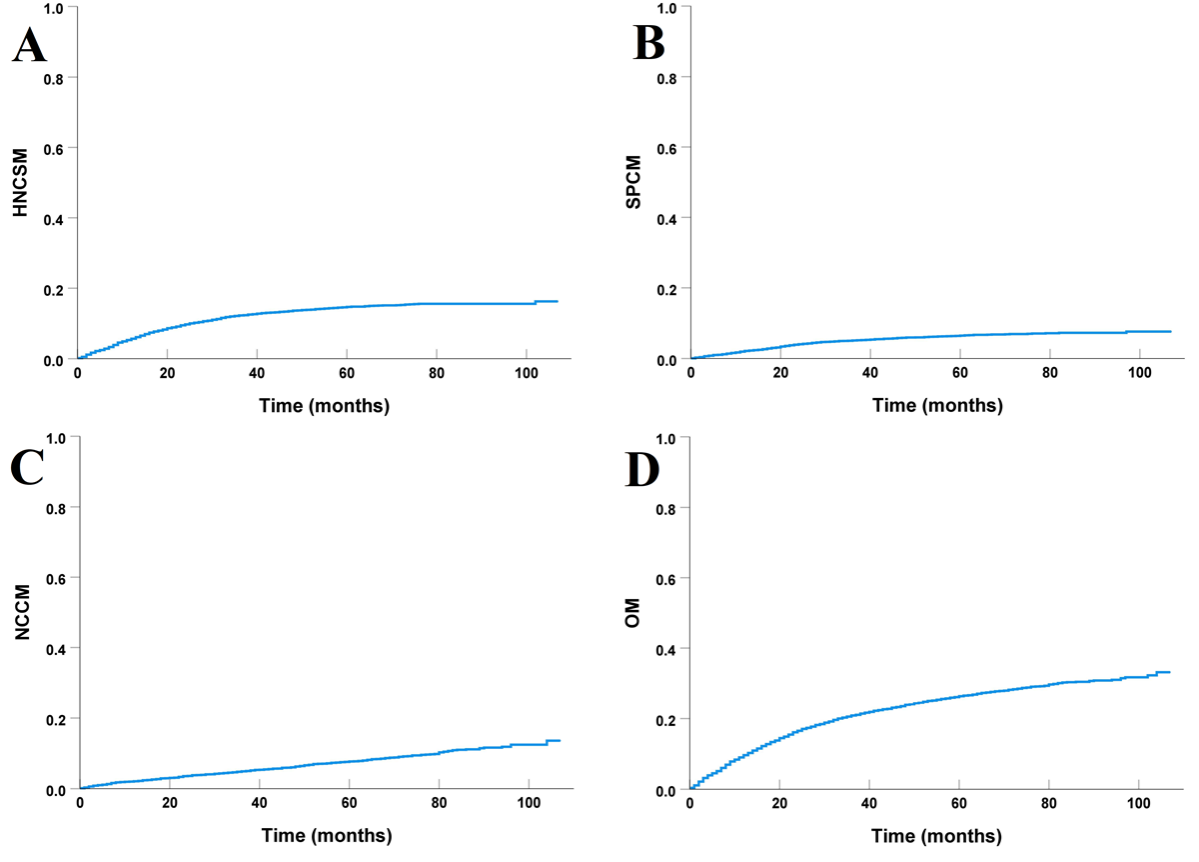
The curves of (A) head and neck cancer–specific mortality, (B) second primary cancer mortality, (C) noncancer-caused mortality, and (D) overall mortality in the entire cohort. HNCSM: head and neck cancer–specific mortality; NCCM: noncancer-caused mortality; OM: overall mortality; SPCM: second primary cancer mortality.

With a median follow-up time of 52 months (range, 0-107 months), a total of 1537 (26.3%) patients died. There were 789 (51.3%), 333 (21.7%), and 415 (27%) patients who died from HNC, second cancer, and noncancer causes, respectively. The survival curves have been listed in Figure 3. The 5-year HNCSM, SPCM, NCCM, and OM were 14.7%, 6.5%, 7.7%, and 26.4%, respectively.

### Competing Mortality

**Figure 4:**
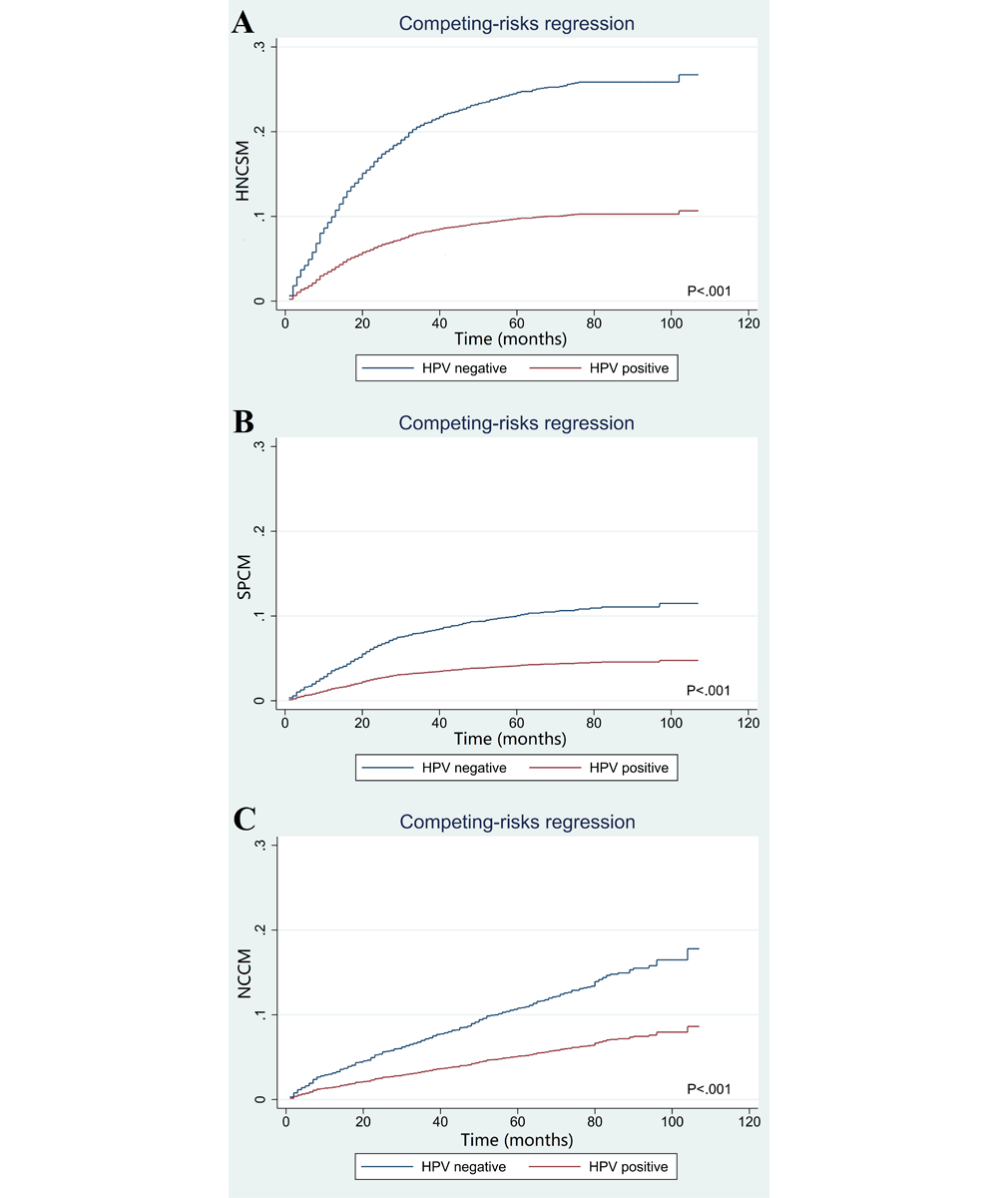
The cumulative incidence of (A) head and neck cancer–specific mortality, (B) second primary cancer mortality, and (C) noncancer-caused mortality by human papillomavirus status using competing-risks regression. HNCSM: head and neck cancer–specific mortality; HPV: human papillomavirus; NCCM: noncancer-caused mortality; SPCM: second primary cancer mortality.

**Figure 5:**
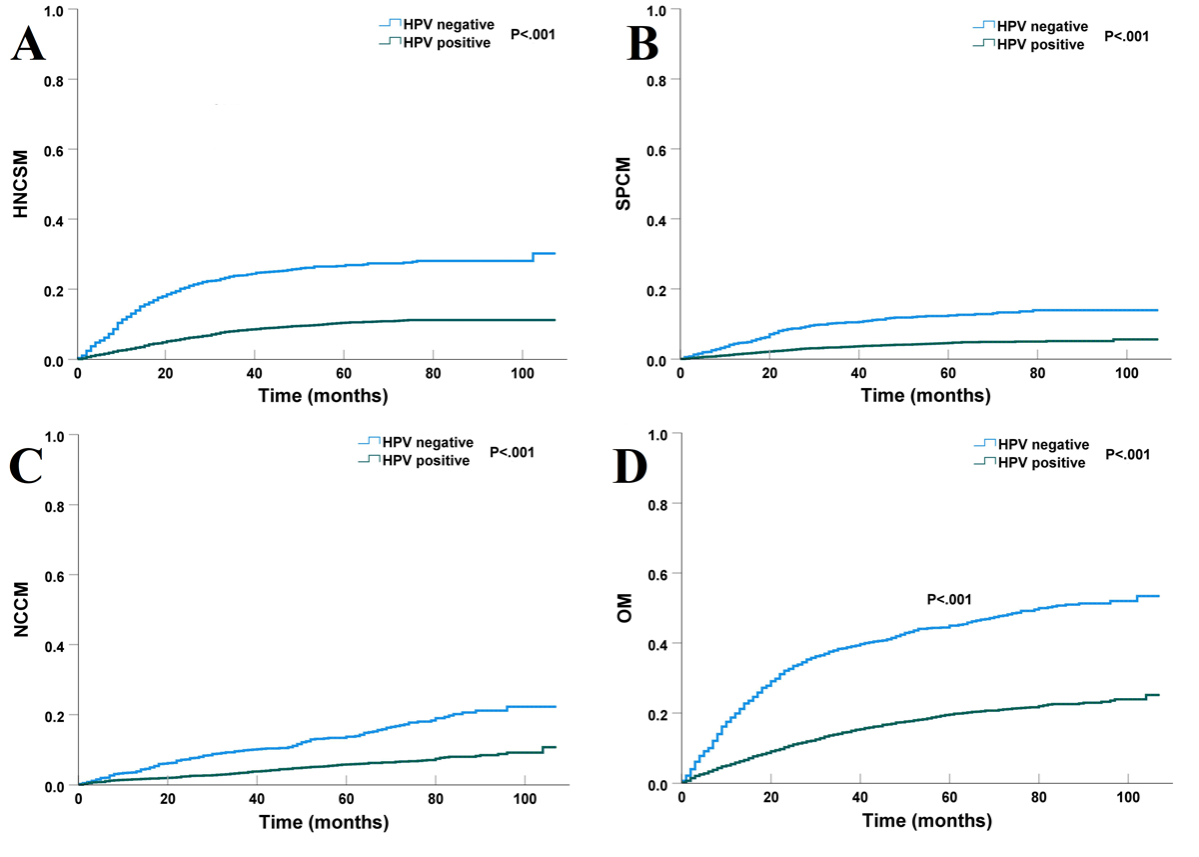
The effect of human papillomavirus status on (A) head and neck cancer–specific survival, (B) overall survival, (C) second primary cancer survival, and (D) noncancer-caused survival. HNCSM: head and neck cancer–specific mortality; HPV: human papillomavirus; NCCM: noncancer-caused mortality; OM: overall mortality; SPCM: second primary cancer mortality.

The cumulative incidence of competing CODs based on HPV status regarding HNCSM, SPCM, and NCCM are listed in Figure 4. Those with HPV-positive disease had a lower cumulative incidence of HNCSM (subdistribution hazard ratio [sHR] 0.362, 95% CI 0.315-0.417; *P*<.001; Figure 4A), SPCM (sHR 0.400; 95% CI 0.321-0.496; *P*<.001; Figure 4B), and NCCM (sHR 0.460; 95% CI 0.378-0.560; *P*<.001; Figure 4C) than those with HPV-negative disease. Using Kaplan-Meier analysis, the results showed that patients who were HPV-negative were more likely to gain experience from second cancer and noncancer causes. The 5-year risk of HNCSM was 26.9% and 10.7% in those with HPV-negative and HPV-positive disease, respectively (*P*<.001, Figure 5A). The 5-year risk of SPCM was 12.4% and 4.6% in those with HPV-negative and HPV-positive disease, respectively (*P*<.001, Figure 5B). The 5-year risk of NCCM of death was 13.7% and 5.8% in those with HPV-negative and HPV-positive disease, respectively (*P*<.001, Figure 5C). The 5-year OM was 26.1% and 10.7% in those with HPV-negative and HPV-positive, respectively (*P*<.001; Figure 5D).

### Fine and Gray Competing-Risks Model

Table 2 shows the sHR and 95% CI for selected covariates using competing-risks regression for different COD. The results of the multivariate analysis showed that the variables related to HNCSM were female, older age, Black, advanced stage, HPV-negative, receipt of surgery, and receipt of radiotherapy (all *P*<.05). Advanced stage, HPV-negative, receipt of surgery, and receipt of radiotherapy were the independent risk factors associated with SPCM (all *P*<.05). Older age, Black race, HPV-negative, receipt of surgery, and receipt of chemotherapy were the independent risk factors associated with NCCM (all *P*<.05). Stratum-specific analyses also showed that patients with HPV-positive tumor had lower HNCSM, SPCM, and NCCM than those with HPV-negative tumors after stratification by different demographic, clinicopathologic, and treatment characteristics (Table S1 in Multimedia Appendix 1).

**Table 2:** Multivariate prognostic analyses using the competing-risks model.

Variables		HNC^a^-specific mortality			Second primary cancer mortality			Noncancer-caused mortality		
		sHR^b^	95% CI	*P* value	sHR	95% CI	*P* value	sHR	95% CI	*P* value
**Gender**										
	Male (reference)	1	N/A^c^	N/A	1	N/A	N/A	1	N/A	N/A
	Female	1.218	1.012-1.467	.04	0.729	0.528-1.005	.05	0.993	0.771-1.279	.96
**Age (years)**										
	<50 (reference)	1	N/A	N/A	1	N/A	N/A	1	N/A	N/A
	50-64	1.497	1.160-1.932	.002	1.231	0.872-1.737	.24	1.251	0.861-1.819	.24
	≥65	2.176	1.665-2.844	<.001	1.26	0.868-1.830	.22	3.139	2.164-4.554	<.001
**Race**										
	White (reference)	1	N/A	N/A	1	N/A	N/A	1	N/A	N/A
	Black	1.525	1.215-1.913	<.001	1.167	0.811-1.677	.41	1.548	1.131-2.120	.006
	Other	1.049	0.721-1.525	.08	0.931	0.523-1.658	.81	1.272	0.787-2.055	.33
**AJCC^d^ stage**										
	I (reference)	1	N/A	N/A	1	N/A	N/A	1	N/A	N/A
	II	2.147	1.117-4.126	.02	1.124	0.435-2.901	.81	0.986	0.575-1.691	.96
	III	2.969	1.637-5.384	<.001	0.403	0.615-3.201	.42	0.895	0.545-1.469	.66
	IVA and IVB	4.515	2.534-8.043	<.001	2.583	1.168-5.713	.02	0.875	0.542-1.412	.59
**Grade**										
	Well differentiated (reference)	1	N/A	N/A	1	N/A	N/A	1	N/A	N/A
	Moderately differentiated	0.797	0.581-1.094	.16	1.083	0.638-1.839	.77	1.059	0.669-1.676	.81
	Poorly or undifferentiated	0.590	0.430-0.810	.001	0.814	0.480-1.381	.45	0.847	0.532-1.351	.49
**HPV^e^ status**										
	Negative (reference)	1	N/A	N/A	1	N/A	N/A	1	N/A	N/A
	Positive	0.439	0.378-0.508	<.001	0.437	0.349-0.547	<.001	0.584	0.472-0.721	<.001
**Surgery**										
	No (reference)	1	N/A	N/A	1	N/A	N/A	1	N/A	N/A
	Yes	0.463	0.389-0.552	<.001	0.524	0.400-0.689	<.001	0.572	0.456-0.716	<.001
**Radiotherapy**										
	No (reference)	1	N/A	N/A	1	N/A	N/A	1	N/A	N/A
	Yes	0.407	0.318-0.524	<.001	0.387	0.267-0.561	<.001	0.932	0.666-1.304	.68
**Chemotherapy**										
	No (reference)	1	N/A	N/A	1	N/A	N/A	1	N/A	N/A
	Yes	0.987	0.801-1.215	.90	1.152	0.834-1.592	.39	0.688	0.525-0.877	.003

^a^HNC: head and neck cancer.

^b^sHR: subdistribution hazard ratio.

^c^N/A: not applicable.

^d^AJCC: American Joint Committee on Cancer.

^e^HPV: human papillomavirus.

## Discussion

### Principal Findings

In this study, we investigated the competing mortality in OPSCC by HPV status using a large cohort. Our results indicated that patients with HPV-negative tumors had a significantly higher risk of HNCSM, SPCM, and NCCM than those with HPV-positive tumors. The main finding of our study was that HPV-positive disease was an independent factor for better survival outcomes across all COD.

HPV status has been confirmed to be an important prognostic and predictive biomarker in OPSCC [12,13]. In recent years, several studies also have explored the relationship between HPV status and the second tumor in OPSCC. Gorphe et al [30] analyzed 888 patients with OPSCC between 2011 and 2020, they found that the 5-year competing risks of a second cancer were 16.1% and 49.9% in those patients who were HPV-positive and HPV-negative, respectively (*P*<.001). In addition, a study from Bosshart et al [31] that included 91 patients also found that HPV-positive had a lower risk of second cancer than those with HPV-negative tumors (14% vs 38.2%). Since the time of COD was only recorded in the SEER database, we could not obtain the exact time of diagnosis of the second primary tumor. However, Culié et al [32] included 340 patients with OPSCC from 14 French hospitals, and they found that patients who were HPV-positive developed low second tumors than patients who were HPV-negative (3.3% vs 11.7%, *P*=.02). However, the COD was similar in the 2 groups.

In our study, we found HNC-related death was the main COD in OPSCC (14.7%), followed by noncancer causes (7.7%), and second cancer (6.5%). Alcohol and tobacco use have been confirmed as risk factors for HNSCC. HPV-related OPSCC cannot be considered in the same field of cancerization as HPV-negative OPSCC and other HNSCCs [2-5]. However, we observed a quite similar distribution of CODs between the 2 groups, with primary cancer as the major COD. Our study suggested that the overall survival pattern of patients with OPSCC depends not only on their clinicopathological characteristics, but also significantly varies based on their age and the presence of comorbidities.

Several previous studies have assessed CODs in patients with HNSCC. They found that age, gender, race or ethnicity, presence of comorbidities, BMI, or type of treatment were the risk factors in the competing COD in patients with HNSCC [22,33-37]. In our study, we analyzed whether the HPV status would influence the COD of patients with OPSCC. A population-based cohort from Denmark was the first to investigate COD by HPV status in 1521 patients with OPSCC [26]. There were 54.3% of patients were HPV-positive and they found that HPV-positive was associated with better survival across all CODs [26]. Lop et al [38] included 423 patients with OPSCC and 12.5% were HPV-positive. They found that patients with HPV-positive disease had better OPSCC-specific survival (*P*<.001) and second primary cancer survival (*P*<.001) than those with HPV-negative tumors. However, noncancer-related causes survival was similar between the 2 groups (*P*=.13). The difference in patient characteristics may affect the distribution of CODs in the previous studies. Those with HPV-positive disease had a significantly lower cumulative incidence of death from all CODs than those with HPV-negative disease. Therefore, the inferior survival in patients with HPV-negative tumors was reflected in the higher risk of HNCSM, SPCM, and NCCM. Patients with HPV-negative OPSCC may benefit from screening for the risk to die of noncancer death or early detection of second cancers. Our study suggests that OPSCC should undergo more different effective therapies, more prevention methods to avoid noncancer deaths, and more screening tools for early detection of the second tumor according to different HPV status. They may benefit from increased research funding.

There were several studies indicating that those with HPV-positive OPSCC have a favorable survival than those with HPV-negative disease regardless of the treatment received [39,40]. In our study, 89.8% of patients were locally advanced-stage OPSCC, we also found that the receipt of treatment was associated with all CODs. The probability of treatment compliance was not recorded in the SEER database. Hess et al [41] found that treatment compliance in patients who were HPV-negative was significantly lower than in those with patients who were HPV-positive. Therefore, treatment compliance by HPV status might also have a negative effect on cancer-specific survival in OPSCC.

Screening for secondary cancer has an important effect in reducing mortality, especially for the HPV-negative population with a higher level of tobacco use [42]. Our data indicate that there were 51.3% and 21.7% patients who died from HNC and second cancer. Therefore, more frequent follow-ups should be performed to increase the detection of recurrence and secondary cancers. Moreover, our study shows an increased risk of SPCM, and NCCM in patients who were HPV-positive than in those with HPV-negative tumors. Our finding is important because it adds to the current knowledge regarding the differences in CODs among HPV status and supports the need to individualize follow-ups according to the HPV status.

In our study, 415 patients died of noncancer-related diseases, and 127 (30.6%) died from heart disease. In our study, patients with HPV-positive OPSCC tend to be younger, White, and male. Previous studies also showed that women of the Black race had a higher risk of cardiovascular disease in the general population and were cancer survivors [43,44]. According to the differences regarding epidemiological characteristics by HPV status may affect the burden of comorbidity, thus affecting the NCCM.

HPV-related tumors have a significant burden on health insurance [45]. While the HPV vaccination has been shown to prevent cervical cancer, HPV vaccination also has the potential to prevent a significant proportion of HPV-related OPSCC. We should note that 84.1% of patients in our study were male, and 85.6% were aged 50 years or older. The vaccine only prevents new HPV infections and does not treat existing infections [46]. HPV vaccination is generally not recommended for those older than age 26 years who are unvaccinated, recent guidelines have a permissive recommendation for HPV vaccination in unvaccinated adults aged 27-45 years [46]. Additionally, 47.7% of patients continued to smoke after their OPSCC diagnosis [47]. Therefore, current smoking status should be monitored throughout the treatment course of OPSCC, and smoking-cessation assistance should be provided as necessary, especially for patients with negative HPV status and a history of smoking. Therefore, further studies are needed to verify the efficacy of HPV vaccination in reducing the incidence of OPSCC and to investigate the effect of smoking cessation on various COD in patients with OPSCC.

We acknowledge certain limitations in our study. First, retrospective studies have inherent weaknesses of selection bias. Second, the lack of data regarding other sexually transmitted infections limits the interpretation of our data because behaviors that put people at risk for HPV also increase their risk for other sexually transmitted infections. Third, the lack of comorbidity data also limits the interpretation of our data. Of note, HNCSM, SPCM, and NCCM data were available and were used in this study. Fourth, the consumption of smoking and alcohol was not recorded in the SEER database. The high rates of HPV-positive OPSCC in our study could potentially be attributed to the low prevalence of smoking habits and differences in sexual behavior when compared with other geographic regions with a low incidence of HPV-positive OPSCC [37,48]. This difference in the incidence of HPV-positive tumors could limit the generalization of our findings to regions with a low incidence of HPV-positive tumors and high levels of tobacco and alcohol use. Moreover, the median follow-up time was 52 months in our study and second cancer or noncancer death is likely to develop after this delay. Finally, the SEER does not have specific records on HPV status detection methods. However, a previous study showed that the overall HPV status (positive or negative) had high degrees of accuracy in the SEER database [49]. Further research addressing these limitations could provide a more comprehensive understanding of the relationship between HPV status and competing mortality in patients with OPSCC.

### Conclusions

In conclusion, our study suggests that HPV-positive OPSCC has a lower NCSM, SPCM, and NCCM as compared to those with HPV-negative OPSCC. HPV positivity is a favorable prognostic factor in the context of overcoming cancer as well as in terms of reducing the risk of other CODs in OPSCC. These disparities have direct implications on clinical trials aiming to define future therapeutic standards in clinical practice, including treatment intensification or de-escalation according to response rates, investigation of adjuvant treatment after complete response, or follow-up strategies involving imaging modalities and duration. Moreover, medical interventions tailored to the intercurrent CODs may independently provide survival benefits in addition to cancer treatments.

## Appendix

Multimedia Appendix 1 Table S1. Stratum-specific analyses to determine the effect of HPV status according to different demography and clinicopathological characteristics using the Fine and Gray competing-risks model.

## Abbreviations

AJCC: American Joint Committee on Cancer
COD: cause of death
HNC: head and neck cancer
HNCSM: head and neck cancer–specific mortality
HNSCC: head and neck squamous cell carcinoma
HPV: human papillomavirus
NCCM: noncancer-caused mortality
OM: overall mortality
OPSCC: oropharyngeal squamous cell carcinoma
SEER: Surveillance, Epidemiology, and End Result
sHR: subdistribution hazard ratio
SPCM: second primary cancer mortality

## Data Availability

The data set used in the data analysis is publicly available.
