# Supplementary material for: Cause of Death in Patients with Oropharyngeal Carcinoma by Human Papillomavirus Status: Comparative Data Analysis
Source: JMIR Public Health Surveill. 2023 Aug 29;9:e47579. doi: 10.2196/47579 (PMC10498314; doi:10.2196/47579)
Supplement: Multimedia Appendix 1 [file publichealth_v9i1e47579_app1.docx]

**Multimedia Appendix Table S1.** Stratum-specific analyses to determine the effect of HPV status according to different demography and clinicopathological characteristics using the Fine and Gray competing-risks model.

| Variables | HNC-specific mortality | | | Second primary cancer mortality | | | Noncancer-caused mortality | | |
| --- | --- | --- | --- | --- | --- | --- | --- | --- | --- |
|  | sHR | 95%CI | P | sHR | 95%CI | P | sHR | 95%CI | P |
| Gender |  |  |  |  |  |  |  |  |  |
| Male | 0.439 | 0.374-0.518 | <.001 | 0.493 | 0.387-0.627 | <.001 | 0.606 | 0.478-0.768 | <.001 |
| Female | 0.393 | 0.278-0.555 | <.001 | 0.167 | 0.739-0.377 | <.001 | 0.411 | 0.254-0.666 | <.001 |
| Age (years) |  |  |  |  |  |  |  |  |  |
| <50 | 0.415 | 0.248-0.696 | 0.001 | 0.271 | 0.141-0.521 | <.001 | 0.246 | 0.118-0.512 | <.001 |
| 50-64 | 0.381 | 0.311-0.466 | <.001 | 0.471 | 0.358-0.628 | <.001 | 0.509 | 0.373-0.964 | <.001 |
| ≥65 | 0.511 | 0.405-0.645 | <.001 | 0.461 | 0.301-0.706 | <.001 | 0.721 | 0.535-0.972 | .032 |
| Race |  |  |  |  |  |  |  |  |  |
| White | 0.465 | 0.393-0.546 | <.001 | 0.427 | 0.337-0.542 | <.001 | 0.553 | 0.440-0.696 | <.001 |
| Black | 0.281 | 0.172-0.458 | <.001 | 0.522 | 0.250-1.090 | .084 | 0.732 | 0.394-1.359 | .324 |
| Other | 0.355 | 0.150-0.842 | .019 | 0.673 | 0.200-2.266 | .524 | 0.628 | 0.229-1.727 | .369 |
| AJCC stage |  |  |  |  |  |  |  |  |  |
| I | 0.769 | 0.226-2.616 | .675 | 0.639 | 0.102-3.970 | .631 | 0.341 | 0.134-0.866 | .024 |
| II | 0.222 | 0.108-0.456 | <.001 | 0.110 | 0.025-0.466 | .003 | 0.590 | 0.281-1.239 | .164 |
| III | 0.294 | 0.203-0.425 | <.001 | 0.482 | 0.264-0.879 | .017 | 0.785 | 0.491-1.241 | .302 |
| IVA and IVB | 0.471 | 0.399-0.557 | <.001 | 0.405 | 0.351-0.579 | <.001 | 0.536 | 0.413-0.696 | <.001 |
| Grade |  |  |  |  |  |  |  |  |  |
| Well differentiated | 0.385 | 0.202-0.734 | .004 | 0.207 | 0.049-0.875 | .032 | 1.289 | 0.567-2.930 | .543 |
| Moderately differentiated | 0.400 | 0.322-0.496 | <.001 | 0.374 | 0.267-0.523 | <.001 | 0.547 | 0.403-0.743 | <.001 |
| Poorly/undifferentiated | 0.464 | 0.373-0.577 | <.001 | 0.559 | 0.398-0.786 | .001 | 0.543 | 0.403-0.731 | <.001 |
| Surgery |  |  |  |  |  |  |  |  |  |
| No | 0.458 | 0.386-0.545 | <.001 | 0.489 | 0.377-0.634 | <.001 | 0.616 | 0.477-0.795 | <.001 |
| Yes | 0.373 | 0.284-0.493 | <.001 | 0.355 | 0.233-0.541 | <.001 | 0.475 | 0.329-0.685 | <.001 |
| Radiotherapy |  |  |  |  |  |  |  |  |  |
| No | 0.494 | 0.335-0.727 | <.001 | 0.676 | 0.381-1.191 | .175 | 0.735 | 0.431-1.254 | .259 |
| Yes | 0.423 | 0.362-0.496 | <.001 | 0.406 | 0.320-0.515 | <.001 | 0.545 | 0.435-0.683 | <.001 |
| Chemotherapy |  |  |  |  |  |  |  |  |  |
| No | 0.461 | 0.343-0.618 | <.001 | 0.597 | 0.374-0.953 | .031 | 0.488 | 0.337-0.705 | <.001 |
| Yes | 0.422 | 0.356-0.500 | <.001 | 0.410 | 0.319-0.528 | <.001 | 0.626 | 0.483-0.811 | <.001 |

AJCC: American Joint Committee on Cancer; CI: confidence interval; HNC: head and neck cancer; HPV: human papillomavirus; sHR: subdistribution hazard ratio.
